# A Cross-Sectional Analysis of Dietary Patterns in Healthy Adolescents: Energy Balance, Nutrient Intake, Body Mass, and Serum Marker Alterations

**DOI:** 10.3390/children10101714

**Published:** 2023-10-22

**Authors:** Ștefan Adrian Martin, Roxana Maria Martin-Hadmaș

**Affiliations:** 1 Department of Physiology, Center for Advanced Medical and Pharmaceutical Research, “George Emil Palade” University of Medicine, Pharmacy, Science and Technology of Târgu Mureș, Gheorghe Marinescu 38, 540139 Târgu Mures, Romania; 2 Department of Community Nutrition and Food Safety, “George Emil Palade” University of Medicine, Pharmacy, Science and Technology of Târgu Mureș, Gheorghe Marinescu 38, 540139 Târgu Mureș, Romania; roxana.hadmas@umfst.ro

**Keywords:** diet, child/children, adolescent, nutrient, inflammatory markers

## Abstract

(1) Background: With shifts in daily dietary habits, concerns have arisen regarding potential impacts on metabolic health. This study sought to investigate the interplay between nutrient intake and its effects on the anthropometric and inflammatory profiles of young individuals. (2) Methods: Our approach examined the interrelation of caloric, macronutrient, and vitamin intakes with inflammatory markers, serum cholesterol, triglycerides, and other key metrics. The impact of these factors on body mass and inflammation was evaluated. (3) Results: This study found that while increased caloric intake corresponded to a rise in body fat mass, it did not significantly alter body weight, total protein, or fat profile. A dominant carbohydrate intake negatively correlated with vitamin B consumption. Interestingly, only vitamin K showcased a direct association with IL-6, while IL-8 remained unassociated with dietary intake and body mass metrics. (4) Conclusions: Dietary intake undeniably influences nutrient consumption and subsequently affects body mass metrics. Though an escalation in body fat mass was evident with increased food intake, the relationship between vitamins and inflammatory markers, based on macronutrient and caloric intake, remains inconclusive. The findings point to the potential regulatory roles of proteins and select vitamins in inflammation, emphasizing the need for deeper longitudinal studies to further validate these connections.

## 1. Introduction

In pediatric research, it is important to differentiate between children’s energy needs and their daily nutritional requirements. While numerous studies on children’s health reference “daily caloric intake” as an indicator of energy consumption [1], it is worth noting that some investigations predominantly focus on calories without a comprehensive analysis of macro- and micronutrient intake. Subsequent studies [2,3] have explored both energy intake and overall nutrition among the younger demographic. Notably, the results show marked differences influenced by geographical location, regional specifics, and sociodevelopmental stages [4]. This highlights the need for a holistic perspective when assessing children’s dietary patterns and nutritional well-being.

Research has delved into the relationship between energy intake, macronutrient consumption, and body weight. Some studies suggest that carbohydrates and fats might increase the risk of obesity more than proteins [5]. In pediatric research, there is often a prevailing hypothesis that emphasizes the strong connection between a child’s dietary habits and their developmental growth. While energy intake is important, it is not the only consideration. Even with adequate caloric intake, deficiencies in specific nutrients can negatively impact cellular metabolism, hormone secretion, body mass growth, and immune function [6,7].

Recent studies have indicated that consumption of heavily processed foods can lead to reduced nutrient intake, while simultaneously raising caloric consumption [8]. Such excessive caloric intake can yield both nutritional and pathophysiological ramifications in children, potentially manifesting as obesity or, paradoxically, malnutrition [9]. Investigations centered around immunomodulatory adipokines like interleukin 2 (IL-2), interleukin 6 (IL-6), and interleukin 8 (IL-8) have an associated increased body weight with heightened inflammatory marker responses [9,10,11,12,13]. Furthermore, overweight individuals with these elevated markers often show a decreased daily intake of vitamins A, C, and E [14]. On a molecular level, vitamin C has been found to limit extracellular-signal-related kinase phosphorylation, while vitamin D can suppress IL-6 production and boost protein expression [15]. Vitamin E, on the other hand, may reduce the likelihood of increased inflammatory markers [16]. Nevertheless, the data on the effects of other micronutrients remain less extensive [17].

The relationship between micronutrient intake and elevated serum inflammatory markers is not fully understood. Moreover, only a handful of studies have delved into the correlation among nutrient intake, changes in body metrics, and inflammatory reactions in the younger demographic. It remains ambiguous whether micronutrients directly influence inflammation or if their levels are a byproduct of dietary decisions, changes in body composition, or pre-existing inflammatory conditions. It is imperative to recognize that a limited dietary intake can result in lowered micronutrient levels, leading to nutritional shortfalls. These deficiencies are often tied to diets that lack crucial amino acids, adequate unsaturated fats, and complex carbohydrates. Against this backdrop, our study is designed to scrutinize the intake of calories, macronutrients, and micronutrients, while concurrently examining the interplay between dietary habits; body metric alterations; and changes in IL-6, IL-8, proteins, and fats in the blood of healthy young individuals.

## 2. Materials and Methods

In the present cross-sectional study, we examined the relationship between growth measurements and daily dietary intake with serum concentrations of IL-6 and IL-8. We also assessed blood cholesterol, triglycerides, total proteins, and creatine levels.

This research methodology and its procedures were carried out at the Advanced Medical and Pharmaceutical Research Center (CCAMF), a branch of ”George Emil Palade” University of Medicine, Pharmacy, Science, and Technology of Targu Mures. We conducted our study following the guidelines set by the University’s Ethical Committee (Approval No. 259/14.11.2018).

### 2.1. Study Period and Study Sample

**Figure 1:**
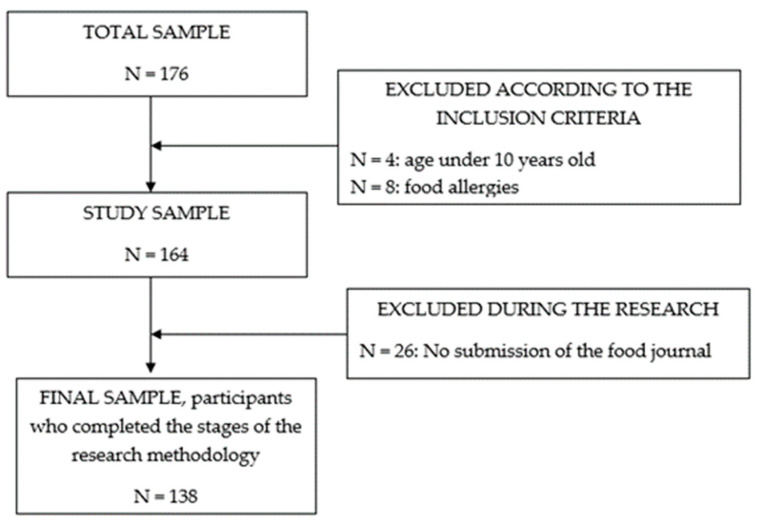
Flow chart of the study sample.

This study, conducted from September 2020 to March 2021, involved 138 participants aged 10 to 16 years, irrespective of their body weight. To be included, participants needed to be within this age range, belong to any gender, and have no history of chronic illnesses, congenital anomalies, food allergies, or chronic and acute respiratory infections, as detailed in Figure 1.

All research activities were thoroughly discussed and proceeded with informed consent. Caregivers oversaw each test’s implementation, and participants were given the freedom to opt out of this study, provided they gave a 2-day notice.

### 2.2. Applied Tests

In a single lab session, participants underwent anthropometric measurements and had their basal metabolic rate (BMR) measured. Subsequently, venous blood samples were drawn from each participant.

#### 2.2.1. Anthropometric Measurements

To evaluate each participant’s physical development, we captured three consistent measurements of body weight, height, and total mass, pausing fifteen seconds between each. For optimal accuracy (ensuring a <1.5% deviation), we used average values. We recorded body weight in kilograms (kg) using the ADE GmbH M304040-01 (Hamburg, Germany), asking participants to wear minimal clothing and stand straight. Their height was measured similarly using the ADE GmbH M304040-01 stadiometer.

We then analyzed developmental markers, setting benchmarks with weight-for-age (%) and height-for-age (%) percentiles. We also calculated the BMI for age percentile and the BMI for age Z score. We classified values between 5% and 95% as within the normal range. Anything below 5% was considered below and values above 95% were considered above the normal range. We processed BMI data using software from the Centers for Disease Control and Prevention [18].

Lastly, to ascertain body composition, we measured skinfold thickness at seven distinct areas using the UK’s HARPENDEN professional skinfold caliper. Utilizing the Durning–Womersley formula, which accounts for age, gender, and an average of four skinfold measurements, we derived both lean body mass and fat mass in terms of kilograms and as a percentage of total body mass [19].

#### 2.2.2. Basal Metabolic Rate Measurement

We used the Cortex Metalyzer 3B-R3 equipment (Cortex Medical, Leipzig, Germany) and the MetaSoft v3 software, after calibration with known O_2_ (15%) and CO_2_ (5%) concentrations. The daily theoretical energy expenditure was calculated using the Harris–Benedict equation, as implemented in the MetaSoft v3 software. Oxygen uptake (VO_2_, mL/min), carbon dioxide production (VCO_2_, mL/min), and energy expenditure (EE, kcal/min/hour/day) were assessed in a supine position using a Basal Metabolic Rate (BMR) protocol. Each assessment lasted between 15 and 20 min and was followed by a 15 min rest period before a subsequent measurement.

#### 2.2.3. Nutritional Analysis

Guardians kept a food journal spanning seven days to capture the dietary patterns, dedicating an average of 15 min daily. Within this timeframe, they provided in-depth details such as food types, serving sizes, quantities, and the timing of meals. We emphasized to both participants and their guardians the importance of retaining their usual dietary habits without introducing new foods or altering existing habits from the past 90 days.

We then analyzed the documented food items using the United States Department of Agriculture’s Food Composition Databases [20]. This review enabled us to ascertain the caloric content (kcal per gram) of each item, along with daily carbohydrate (g), protein (g), and fat (g) intakes. We further evaluated the cholesterol (mg) in each item and broke down the fat content into saturated fats (g), monounsaturated fats (g), polyunsaturated fats (g), and trans fats (g). We also determined the amounts of simple carbohydrates (g) and dietary fiber (g) in the foods. From the data, we calculated the daily intake of vital nutrients such as folic acid (µg), vitamin A (µg), vitamin C (mg), vitamin B12 (mg), vitamin B1 (mg), vitamin B6 (mg), vitamin K (µg), and vitamin E (mg).

#### 2.2.4. Blood Samples

Following a 12 h overnight fast, every participant supplied a venous blood sample. For the two days leading up to the sample collection, participants were advised to limit their physical activity, not exceeding 30 min of mild exercise. Once acquired, the samples underwent centrifugation at 100× *g* rotations per minute (RPM). We then transferred the separated components into 12 µL microtubes and stored them at −80 °C awaiting further analysis.

Utilizing the DYNEX DSX AUTOMATED ELISA system, we assessed the serum levels of interleukin 6 (IL-6, pg/mL) and interleukin 8 (IL-8, pg/mL) via an immunoenzymatic technique. Additionally, we gauged the serum quantities of cholesterol (with readings from 80 to 200 mg/dL), creatinine, triglycerides (with readings from 50 to 150 mg/dL), and total proteins (with readings from 66 to 87 g/L) using the Cobas Integra 400 Plus instrument.

### 2.3. Statistical Evaluation

Statistical analyses were conducted using GraphPad Prism 6.0 software. Initially, the normality of our dataset was verified using the D’Agostino-Pearson test. With a set significance level (α) of 0.05, we adjusted for multiple comparisons using the Bonferroni Correction, resulting in a new significance threshold of α = 0.002. This adjustment was based on a hypothetical maximum sample size of 280 individuals, even though our study comprised a sample of 138 participants.

For comparisons between two parameters, we used the Mann–Whitney test, especially when the data did not conform to normal distribution. In contrast, the Spearman r test was applied to determine the strength and direction of relationships between two variables, a decision influenced by the non-parametric nature of our data.

In presenting the results, we chose to display them as medians with their associated range (min to max) due to the non-normal distribution observed in much of our data. Additionally, the coefficient of variation (CV %) was included to highlight the variability within our dataset. We decided not to include average values, as the daily food intake of our participants showed considerable variation.

## 3. Results

Within the study sample, the median age was 13 years, with corresponding measurements of 47 kg for body weight and 158 cm for body height. Of the study sample, 49% were female and 51% were male participants. The weight-for-age percentile was 55%, the height-for-age percentile was 60%, while the BMI for age percentile reached 49.11% as detailed in Table 1.

### 3.1. Caloric Value and Vitamin Intake

The median daily caloric intake stood at 1.861 kcal, ranging from 773.4 to 3.606 kcal. In terms of macronutrients, the median daily intake included 207.6 g (equivalent to 4.41 g/kg) of carbohydrates, 72.53 g (1.53 g/kg) of fats, and 79.2 g (1.66 g/kg) of proteins.

The daily intake of CHO was 830.4 kcal, of which 42.3 g came from simple CHO. Proteins contributed 313.3 kilocalories per day, and fats provided 650.4 kilocalories daily. Within the fat intake, 8.5 g (CV % = 84.5%) was saturated fats, 9.44 g (CV% = 73.76%) was monounsaturated fats, and 4.03 g (CV% = 93.85%) was polyunsaturated fats. Table 2 provides additional information on macro- and micronutrient intake.

### 3.2. The Relationship between Macronutrients and Vitamins

Protein intake exhibited significant positive correlations with fat-soluble vitamins, such as vitamin A (*p* = 0.0001, r = 0.45), vitamin E (*p* = 0.0001, r = 0.56), and vitamin K (*p* = 0.0001, r = 0.39), as well as two water-soluble vitamins, namely vitamin B1 (*p* = 0.0001, r = 0.61) and vitamin B12 (*p* = 0.0001, r = 0.59). Our findings revealed analogous outcomes concerning fat intake, whereas carbohydrate consumption exhibited a negative correlation with vitamin intake (*p* < 0.002), as further detailed in Table 3.

We further obtained correlations between vitamins and dietary components. Associations between saturated fats and Vitamin B1 (*p* = 0.001, r = −0.53) as well as Vitamin E (*p* = 0.0019, r = 0.30) were observed. In contrast, other fat-soluble and water-soluble vitamins, namely vitamins A, D, K, C, B2, B6, and B12, and folic acid, did not display significant correlations (*p* > 0.002). Similarly, trans fats exhibited positive correlations with vitamin K, vitamin E (*p* = 0.0001), as well as vitamins B1, B6, and B12 (*p* = 0.0001), but not with vitamins A and C and folic acid (*p* > 0.002).

The consumption of simple carbohydrates was linked to fat-soluble vitamins, specifically vitamin K (*p* = 0.001, r = −0.49) and E (*p* = 0.0001, r = −0.53), as well as several water-soluble vitamins: B1 (*p* = 0.001, r = −0.40), B6 (*p* = 0.001, r = −0.43), and folic acid (*p* = 0.001, r = −0.38). In contrast, there were no significant correlations between the consumption of simple carbohydrates and vitamins B12, C, and A (*p* > 0.002).

### 3.3. The Relationship between Energy Needs, Caloric, and Vitamin Intake

The median daily caloric intake was 1.861 kcal, ranging from a minimum of 773.4 kcal to a maximum of 3.606 kcal. In contrast, the theoretical daily energy expenditure was 1.387 kcal (ranging from 411 to 1.915 kcal/day, min to max), and the BMR was 1.704 kcal/day (with a range of 1.461 to 2.520 kcal/day, min to max).

There was a significant difference between the theoretical daily energy expenditure and the actual daily caloric intake (*p* = 0.0001, Mann–Whitney U = 3444). However, the BMR value was not significantly different from the daily caloric intake (*p* = 0.0468, Mann–Whitney U = 6012). The daily caloric intake was significantly correlated with vitamin intake, particularly with water-soluble vitamins, including vitamins B1 (*p* = 0.001, r = 0.61), B6 (*p* = 0.001, r = 0.58), and B12 (*p* = 0.001, r = 0.57), and certain fat-soluble vitamins, such as vitamin K (*p* = 0.001, r = 0.45) and vitamin E (*p* = 0.001, r = 0.57). However, vitamin C, vitamin A, and folic acid failed to correlate with the daily caloric intake (*p* > 0.002).

### 3.4. Serum Results, and Changes in Vitamin Intake

The levels of IL-6 ranged from 0.14 to 5.98 pg/mL, with a median value of 1.38 pg/mL, while IL-8 levels were in the range of 0.72 to 38.9 pg/mL, with a median value of 6.84 pg/mL. Notably, there was no observed relationship between the levels of both fat-soluble and water-soluble vitamins, except for vitamin K, which exhibited a significant correlation with IL-6 levels (*p* = 0.0003, r = −0.368). However, the intake of vitamins did not show a significant correlation with serum IL-8 levels (*p* > 0.002), as further detailed in Table 4.

The intake of protein showed a pronounced positive relationship with total fat consumption (*p* = 0.0001, r = 0.738), which in turn was associated with serum cholesterol levels (*p* = 0.0001, r = 0.85). Furthermore, protein intake demonstrated secondary correlations with B complex vitamins (*p* = 0.0001) and daily fat-soluble vitamins (*p* = 0.0001). The sum of skinfold measurements was correlated with body fat mass, while no other correlation was obtained, as further detailed in Table 5.

## 4. Discussion

Following our study’s approach, we examined caloric, macronutrient, and vitamin intake, alongside levels of IL-6, IL-8, total proteins, cholesterol, triglycerides, and serum creatine, to understand their interplay. From our findings, it is evident that an increase in body fat mass resulting from heightened caloric consumption can lead to altered inflammatory states and irregularities in lipid and protein levels. These outcomes are due to excessive caloric and macronutrient consumption. Yet, it is noteworthy that although dietary intake was linked with body mass, it did not significantly correlate with changes in body weight, total proteins, or fat profile.

From our observations, current daily eating habits have seen considerable changes. Food intake is characterized by deficient protein consumption, with a heightened dependency on carbohydrates and saturated fats. These dietary changes not only lead to reduced nutrient intake but also amplify the risk of metabolic and other health issues.

### 4.1. Caloric, Macronutrient, and Vitamin Intake

In the current study, daily caloric intake did not demonstrate a significant association with variations in body weight. This implies that the dietary composition may exert a greater influence on body mass fluctuations than caloric intake alone. This observation is consistent with other research, such as the study by Benton et al. [21], which recommended reduced calorie intake as an effective approach for weight management. Surprisingly, our data highlighted a theoretical caloric intake of 1386 kcal, with the BMR value at 1704 kcal/day, and an actual mean intake of 1861 kcal/day. These results contest the prevailing understanding, implying that even when caloric intake is lower than energy expenditure, an imbalanced nutrient profile could lead to an increase in adipose tissue accumulation, potentially influencing overall body weight [22].

Our findings indicated a predominant consumption of carbohydrates, while the intake of proteins and fats closely adhered to age-specific guidelines. Elizabeth et al.’s research [23] postulated that processed foods, being nutrient-deficient, could exert a profound influence on caloric and nutrient intake. A similar statement was made by Kiani, regarding the prevention of macro- and micronutrient deficiency [24,25]. This observation may provide insight into the predominance of carbohydrate-focused diets in our study cohort. Moreover, we identified that a surge in daily caloric intake was predominantly associated with carbohydrate consumption, characterized by an average daily intake of 42.9 g of simple carbohydrates. Clemente-Suárez [26] stated several changes brought by the consumption of simple carbohydrates, among which the main changes refer to the development and prognosis of metabolic disease.

Research published by Halbesma et al. and Hooper et al. [27,28], involving vast cohorts, suggested a correlation between protein intake and cardiovascular risks among adults. Contrarily, our findings seem to deviate from this mainstream perspective. Keller’s review [29] and Qi’s paper [30] accentuated the critical role of serum proteins, their availability, and the subsequent implications for overall nutritional health. Notably, a decline in protein status was pegged to malnutrition, dietary missteps, and inflammatory markers. Our dataset indicated sub-optimal protein intake, which intriguingly correlated with less lean mass and with lower vitamin consumption. Morais et al.’s findings [31] resonated with our observations, underscoring the correlation between protein intake and the risk of malnutrition. Yet, several studies have linked the risk of malnutrition to inadequate breastfeeding during early childhood [32], further underscoring the need for longitudinal research on this topic.

Our research also revealed that protein consumption was intricately linked with the intake of various vitamins, leading us to hypothesize that higher protein intake might trigger an uptick in fat and, notably, saturated fat intake. Proteins, in their interaction with cellular processes, manifest a broad spectrum of effects. Nagishi et al. [33] determined that a regular to high protein intake can reduce the risk of mortality, especially when derived from specific plant protein sources. Other scholarly endeavors [34] have delved into the interplay between protein intake, its sources, and the modulation of zinc absorption. While our study did not corroborate this aspect conclusively, we concur with the broader scientific consensus that other dietary components might modulate zinc absorption, subsequently influencing protein levels [34].

In prior research, Carr et al. [35] established the involvement of vitamin C in oxidative protein damage. Yet, when it comes to its association with cellular function, vitamin C’s interaction with protein intake remains less understood, with sparse data connecting vitamin C to protein intake [36] in comparison to its well-documented role in oxidative stress. Additionally, based on findings by Pravst et al. [37], protein intake should exhibit a correlation with folate consumption. Their research emphasized the need for a greater intake of foods rich in folate, aligning with previous studies that associated folate with protein metabolism and availability [38]. Given our study sample’s limited protein intake, it is evident that this led to a reduced consumption of certain micronutrients, which may play a role in anthropometric development, as proved in our published paper [39].

Fat intake presented a robust association with caloric intake, serum cholesterol, and triglyceride levels. However, regarding micronutrients, fat intake was not significantly linked with folic acid consumption but was correlated with the average intake of fat-soluble vitamins. It is imperative to recognize the contributions of vitamins A, D, E, and K concerning essential bodily functions like vision, immunity, skin and bone health, reproduction, antioxidation, and hemostasis [40,41]. Their presence and function are influenced by dietary patterns; processed foods, for instance, might influence vitamin absorption and cellular activity. Our data highlighted a substantial consumption of saturated and trans fats, which seemingly limited the availability of fat-soluble vitamins, similar to Souza et al.’s [42] paper. Further, the interplay between fats and vitamins like K, E, and B1–B6 indicates a connection with protein availability, especially when derived from processed foods. A similar statement was made by Mascolo and Vernì [43] who approached protein intake and Vitamin B6 status, which was strongly related.

### 4.2. Vitamins and Blood Sample Alterations

Contrary to expectations, our sample did not show a clear relationship between inflammatory markers and dietary intake. However, the association between IL-6 and vitamin consumption was noteworthy. Elevated inflammatory markers were correlated with vitamin K levels. While some studies have underscored vitamin B1’s capacity to diminish IL-6 levels, paralleling observations with TNF-α and IL-1β [40], others have highlighted vitamin E’s potential to reduce serum C-reactive protein and IL-6 concentrations [16]. In our research, an increased level of the inflammatory marker was correlated with IL-6, consistent with Shea et al.’s paper [44]. Conversely, the lack of association between vitamin D levels and inflammatory markers was unexpected, especially in light of the findings by Aksan et al. [45], who documented reduced vitamin D levels corresponding to elevated inflammatory markers. Nevertheless, our findings emphasized that elevated non-active body mass might influence inflammatory responses, underscoring the importance of body mass as a risk factor in pediatric and adolescent populations [46].

We found no significant relationship between serum cholesterol and certain fat-soluble vitamins, contrasting findings from Saggini et al. [47]. Concurrently, Zalaket et al. [48] identified a link between vitamin A and plasma HDL cholesterol. Elevated vitamin A intake was associated with diminished HDL cholesterol levels in their research. Additionally, an inverse relationship was observed between serum triglycerides and vitamin B12, which is further linked to protein and fat consumption [49]. Drawing upon Ranasinghe et al. [50], the presence of zinc appears to mitigate serum lipids, potentially decreasing atherosclerosis risks, although this hypothesis remains unverified in younger individuals.

### 4.3. Study Limitations

In interpreting the results of this study, several limitations must be acknowledged. Firstly, the observed emphasis on carbohydrate-centric diets in our sample introduces potential variability, which might not be fully accounted for. Discrepancies between our findings on vitamin D and inflammatory markers, in comparison to previous studies such as Aksan et al. [45], hint at potential methodological or regional differences. Potential confounders like physical activity levels or genetic predispositions were not exhaustively explored. Moreover, the specificity of our sample may restrict the generalizability of the findings to broader populations and, lastly, measurement inaccuracies in self-reported dietary data might introduce potential errors. However, further analyses should extend to gender-specific food intake.

## 5. Conclusions

Our study found that nutrient intake impacts the anthropometric development of young individuals. Specifically, increased carbohydrate intake was inversely related to vitamin B intake. While most inflammatory markers remained unchanged with varying vitamin levels, vitamin K correlated with IL-6. However, IL-8 showed no correlation with food intake and body mass. Our findings emphasize the influence of food intake on nutrient absorption, body mass changes, and potential shifts in inflammatory markers, highlighting the importance of dietary considerations for both obese and normal-weight individuals. While certain proteins and vitamins appear to regulate inflammation, the cross-sectional design of our study means causative relationships cannot be confirmed. Longitudinal studies are recommended for a deeper exploration of these findings.

## Figures and Tables

**Table 1:** Descriptive data regarding the age and the stage of anthropometric development.

Study Parameter	Median (Min to Max)	CV (%)
Age, years old	13 (10 to 16)	12.14
Body Weight, kg	47 (25.4 to 75.5)	22.43
Body Height, cm	158 (129 to 179)	46.6
Fat mass, %	16.7 (6.6 to 39.3)	42.16
Fat-free mass, %	36.2 (34.4 to 43.6)	17.67
The sum of skinfolds, mm	58.75 (11 to 204)	46.65
BMI, kg/m^2^	18.60 (14.10 to 25.30)	11.71
BMI for age Z score	−0.02 (−1.161 to 1.733)	-
BMI for age percentile	49.11 (5.36 to 95.84)	49.51
Weight for age percentile, %	55 (25 to 115)	55.83
Height for age percentile, %	60 (26.75 to 142)	58.89

Legend: Min—minimum; Max—maximum; CV—coefficient of variation; kg—kilograms; cm—centimeters; %—percentage; mm—millimeters; BMI—Body mass index.

**Table 2:** Macronutrient intake, summarizing median values for macronutrients and vitamin consumption over a day period.

Macronutrient		Median (Min to Max)	CV (%)
Macronutrients	Carbohydrates (g/day)	207.6 (100.3 to 393)	29.37
	Proteins (g/day)	79.2 (28.2 to 227.9)	36.63
	Fats (g/day)	72.53 (16.55 to 164.3)	39.80
Vitamins	A (µg/day)	423.6 (83.52 to 2998)	97.77
	C (mg/day)	29.24 (0.6 to 191.9)	87.26
	B1 (mg/day)	0.935 (0.15 to 1.97)	45.57
	B6 (mg/day)	0.98 (0.33 to 3.01)	50.62
	B12 (µg/day)	1.21 (0 to 12.24)	110.45
	K (µg/day)	17.14 (0 to 489.5)	181.96
	E (mg/day)	1.175 (0.13 to 5.6)	83.57
	Folic Acid (µg/day)	7.05 (0 to 386.6)	212.32

Legend: Min—minimum; Max—maximum; g/day—grams per day; pg/mL—picograms per milliliter; mg/dL—milligrams per deciliter; mg/day—milligrams per day; µg/day—micrograms per day.

**Table 3:** Statistical analysis between proteins, fats, carbohydrates, and daily vitamin intake.

Parameter (Median, Min to Max)	Proteins (69.19, 59.71 to 75.4 g/Day)		Fats (72.27, 16.55 to 164.3 g/Day)		Carbohydrates (207.6, 100.3 to 393 g/Day)	
	*p*-Value	*r* Value	*p*-Value	*r* Value	*p*-Value	*r* Value
**Vitamin A** (423.6, 83.52 to 2998 µg/day)	0.0001	0.454	0.0009	0.321	0.0001	−0.374
**Vitamin C ** (29.24, 0.6 to 191.9 mg/day)	0.1381	0.146	0.0001	0.66	0.0677	0.179
**Vitamin B12** (1.21, 0 to 12.24 µg/day)	0.0001	0.597	0.0001	0.552	0.0001	−0.522
**Vitamin B1** (0.935, 0.15 to 1.97 mg/day)	0.0001	0.611	0.0001	0.517	0.0001	−0.535
**Vitamin B6** (0.98, 0.33 to 3.01 mg/day)	0.0001	0.699	0.0001	0.401	0.0001	−0.65
**Vitamin K** (17.14, 0 to 489.5 µg/day)	0.0001	0.394	0.0001	0.493	0.0008	−0.324
**Vitamin E** (1.175, 0.13 to 5.6 mg/day)	0.0001	0.566	0.0001	0.564	0.0001	−0.454
**Folic Acid** (7.05, 0 to 386.6 µg/day)	0.1266	0.150	0.5693	0.056	0.0042	−0.278

Legend: Min—minimum; Max—maximum; g/day—grams per day; pg/mL—picograms per milliliter; mg/dL—milligrams per deciliter; mg/day—milligrams per day; µg/day—micrograms per day; *p*—probability level; *r*—Pearson/Spearman product-moment correlation coefficient.

**Table 4:** Vitamins and inflammatory markers: inferential analysis.

Parameter (Median, Min to Max)	IL-6 (1.41, 0.05 to 5.98 pg/mL)		IL-8 (7.09, 0.72 to 38.9 pg/mL)	
	*p*-Value	*r* Value	*p*-Value	*r* Value
**Vitamin A** (423.6, 83.52 to 2998 µg/day)	0.156	−0.1475	0.3286	−0.1019
**Vitamin C** (29.24, 0.6 to 191.9 mg/day)	0.0774	−0.1831	0.4269	0.08291
**Vitamin B12** (1.21, 0 to 12.24 µg/day)	0.1969	0.1343	0.9921	0.001042
**Vitamin B1** (0.935, 0.15 to 1.97 mg/day)	0.0101	−0.2642	0.2763	−0.1135
**Vitamin B6** (0.98, 0.33 to 3.01 mg/day)	0.1944	−0.135	0.897	0.01353
**Vitamin K** (17.14, 0 to 489.5 µg/day)	0.0003	−0.368	0.1066	−0.1675
**Vitamin E** (1.175, 0.13 to 5.6 mg/day)	0.0248	−0.2314	0.6247	−0.05111
**Folic Acid** (7.05, 0 to 386.6 µg/day)	0.0829	−0.1798	0.8382	0.02134

Legend: pg/mL—picograms per milliliter; mg/dL—milligrams per deciliter; mg/day—milligrams per day; µg/day—micrograms per day; p—probability level; r—Pearson/Spearman product-moment correlation coefficient.

**Table 5:** Statistical analysis conducted between vitamin intake and blood sample analysis.

Parameter (Median, Min to Max)	Cholesterol (144.7, 91.33 to 245.3 mg/dL)		Total Proteins (69.13, 59.71 to 76.41 g/L)		Triglycerides (49.46, 25.67 to 152.4 mg/dL)		Creatinine (0.58, 0.43 to 0.9 mg/dL)	
	*p*-Value	*r* Value	*p*-Value	*r* Value	*p*-Value	*r* Value	*p*-Value	*r* Value
**Vitamin A** (423.6, 83.52 to 2998 µg/day)	0.032	−0.220	0.0781	−0.182	0.9312	0.009	0.001	0.323
**Vitamin C** (29.24, 0.6 to 191.9 mg/day)	0.769	−0.030	0.1568	−0.147	0.3692	−0.093	0.041	0.210
**Vitamin B12** (1.21, 0 to 12.24 µg/day)	0.097	−0.171	0.5964	−0.055	0.0345	0.218	0.007	0.272
**Vitamin B1** (0.935, 0.15 to 1.97 mg/day)	0.287	−0.110	0.0184	−0.242	0.1885	0.136	0.0635	0.192
**Vitamin B6** (0.98, 0.33 to 3.01 mg/day)	0.493	−0.071	0.1208	−0.161	0.8756	0.016	0.1776	0.140
**Vitamin K** (17.14, 0 to 489.5 µg/day)	0.004	−0.289	0.023	−0.233	0.3686	0.093	0.091	0.175
**Vitamin E** (1.175, 0.13 to 5.6 mg/day)	0.0301	−0.223	0.001	−0.334	0.6668	0.044	0.1211	0.161
**Folic Acid** (7.05, 0 to 386.6 µg/day)	0.487	−0.072	0.018	−0.242	0.0412	−0.211	0.6187	−0.052

Legend: Min—minimum; Max—maximum; g/day—grams per day; pg/mL—picograms per milliliter; mg/dL—milligrams per deciliter; mg/day—milligrams per day; µg/day—micrograms per day; p—probability level; r—Pearson/Spearman product-moment correlation coefficient.

## Data Availability

The data that support the findings of this study are available on request from the corresponding author. The data are not publicly available due to privacy or ethical restrictions.
